# One-year Survey of human enteroviruses from sewage and the factors affecting virus adsorption to the suspended solids

**DOI:** 10.1038/srep31474

**Published:** 2016-08-11

**Authors:** Zexin Tao, Zhongtang Wang, Xiaojuan Lin, Suting Wang, Haiyan Wang, Hiromu Yoshida, Aiqiang Xu, Yanyan Song

**Affiliations:** 1 Academy of Preventive Medicine, Shandong University, Jinan, People’s Republic of China; 2 Shandong Provincial Key Laboratory of Infectious Disease Control and Prevention, Shandong Center for Disease Control and Prevention, Jinan, People’s Republic of China; 3 Department of Radiation Oncology, Shandong’s Key Laboratory of Radiation Oncology, Shandong Cancer Hospital, Jinan, People’s Republic of China; 4 Department of Virology II, National Institute of Infectious Diseases, Tokyo, Japan; 5 School of Public Health, Shandong University, Jinan, People’s Republic of China

## Abstract

This study described the results of environmental enterovirus surveillance conducted in Shandong Province of China in 2013. Altogether 39 sewage samples were collected and 873 enterovirus isolates (including 334 polioviruses) belonging to 22 serotypes were obtained. Echovirus (E) -7, coxsackievirus (CV) -B5, E-11, E-6, and E-3 were the most commonly detected non-polio enterovirus serotypes, and phylogeny of E-7 and CV-B5 was described. The numbers of isolates of different serotypes from sewage supernatant were compared with those from the solids. Interestingly, dramatic divergence was observed between the supernatant and solids origin for the serotypes of E-3 and E-6, which were prone to the solids and supernatant, respectively. A following adsorption test with E-3 and E-6 added sewage specimens confirmed the different preference. Furthermore, the adsorption of Sabin poliovirus type 1 to the solids under different conditions was investigated, and the results showed that acid medium, cold temperature, and high solids concentration facilitated the viral adsorption to the solids, whereas change of virus titer did not influence the proportion of adsorption. These results highlighted the importance of combining the enterovirus isolates from the supernatant and solids together in environmental surveillance so as to better understand the local circulation of different serotypes.

Human enteroviruses (EVs), comprising more than 100 serotypes, belong to the genus *Enterovirus*, family *Picornaviridae*^1^. They are associated with a variety of clinical presentations such as acute flaccid paralysis (AFP), meningitis, encephalitis, cardiac disease, pleurodynia, acute hemorrhagic conjunctivitis, hand-foot-and-mouth disease (HFMD), etc ref. 2.

Environmental surveillance means monitoring EV transmission in human populations by examining specimens (mostly sewage) contaminated by human faeces. It has been conducted as a supplementary method in the Global Polio Eradication Initiative (GPEI), and as the morbidity:infection ratio of poliovirus (PV) infection is very low, environmental surveillance has better sensitivity than the standard AFP surveillance under optimal conditions^3^. Surveillance on sewage has been demonstrated to be an effective approach in investigating local circulation of PV and non-polio enteroviruses (NPEVs)^4^,^5^. Especially in countries with limited disease-based EV surveillance system, data from environmental surveillance are of great public health significance^6^,^7^.

Various techniques have been used for sewage processing, and sewage supernatant was the most frequent source for EV detection^3^. However, EVs adsorb readily to small solid particles suspended in the sewage^8^, and previous investigations on river water had shown that the numbers of virions recovered from the solids even exceeded those recovered from the supernatant^8^. Therefore, as enterovirus environmental surveillance has been regularly conducted in an ever increasing number of enterovirus laboratories throughout the world in recent years, understanding the extent of such adsorption under different conditions is of great significance. It is still unclear whether different serotypes have similar proneness in adsorption to the solid, and this should also be clarified.

In this study, we described the findings in the environmental surveillance in eastern China in 2013. We also explored the adsorption of Sabin PV type 1 (PV-1) to the solids under different pH values, temperatures, viral titers, and the solids concentrations.

## Results

### Surveillance Overview in Shandong, 2013

A total of 39 sewage samples (19 in Jinan city and 20 in Linyi city) were collected from January to December 2013. Supernatant from all 39 samples and the solids from 26 samples were processed and used for EV isolation. The results showed that EVs were detected from all 39 samples with a positive rate of 100%. PVs were detected from 32 samples with a positive rate of 82.1%. Altogether 873 EV isolates (334 PVs and 439 NPEVs) belonging to 22 serotypes were obtained (Table 1). Echovirus (E) -7, coxsackievirus (CV) -B5, E-11, E-6, and E-3 were the most commonly detected NPEV serotypes accounting for 29.67%, 8.82%, 6.76%, 4.58%, and 4.01%, respectively of total isolation. PV-1, -2, and -3 accounted for 16.6%, 8.7%, and 12.9%, respectively of total isolation. They were all Sabin strains with no wild poliovirus (WPV) or vaccine-derived poliovirus (VDPV).

### Close Genetic Relationship within E-7 and CV-B5 Isolates

Phylogenetic analysis was performed on partial VP1 sequences of E-7 and CV-B5 strains from the sewage and those of other reference strains. Considering the high number of E-7 isolates (n = 259), sequences with >99.5% similarities were manually removed.

**Figure 1:**
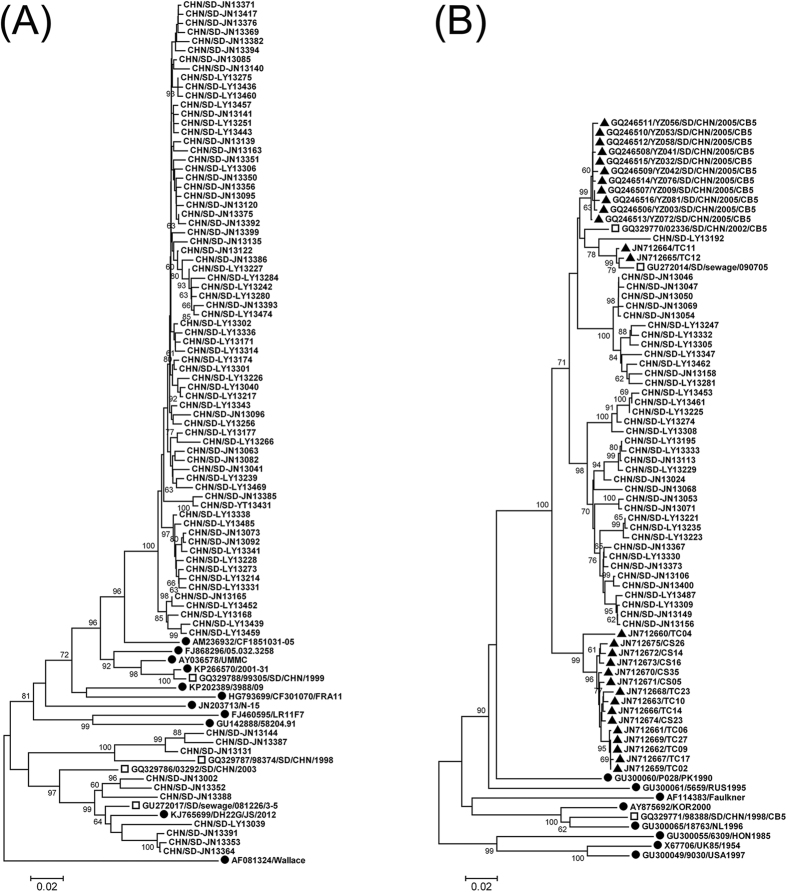
Phylogenetic analysis of E-7 (**A**) and CV-B5 (**B**) based on VP1 sequences. The phylogenetic trees were constructed using Mega, version 4.0, using the NJ method based on 712-nt (positions 2611 to 3322 on strain Wallace) and 685-nt (positions 2611 to 3295 on strain Faulkner) partial VP1 sequences of E-7 and CV-B5, respectively. ●, global reference strains. □, Shandong strains previously isolated from AFP cases or sewage. ▲ in B, CV-B5 strains from two aseptic meningitis outbreaks in Shandong in 2005 and 2009, respectively. The rest are sequences from environmental strains in this study.

Environmental E-7 sequences were grouped into two clusters (Fig. 1A). Both clusters contained isolates from Jinan and Linyi. E-7 isolates in the major cluster had close relationship among themselves with less than 4.5% pairwise genetic diversity. Environmental CV-B5 sequences formed into a major cluster together with sequences from two aseptic meningitis outbreaks in 2005 and 2009, respectively (Fig. 1B) (Chen *et al*.^9^). These environmental sequences had less than 8.8% nucleotide diversity with each other.

### Similar Isolation between the Supernatant and Solids Origin Except for E-3 and E-6

As mentioned above, 26 specimens (12 from Jinan and 14 from Linyi) were used for the comparison between the two origins. Both solids and supernatant of these 26 specimens were processed and EV isolation was performed and statistically compared in matched pair. The results showed that a total of 717 EV strains of 20 serotypes were recovered from the solids and supernatant of these 26 sewage samples (Table 1). Generally, the number of isolates from the solids resembles with that from the supernatant for most serotypes, except for E-3 and E-6. Wilcoxon signed ranks test on 3 PV types and 5 most common NPEV serotypes (E-7, CV-B5, E-11, E-6, and E-3) was conducted. Significant difference was observed for the serotypes E-3 and E-6 (*P *< 0.05), indicating E-3 in the sewage preferred to be adsorbed to the solids than staying in the supernatant, and E-6 was the opposite.

**Figure 2:**
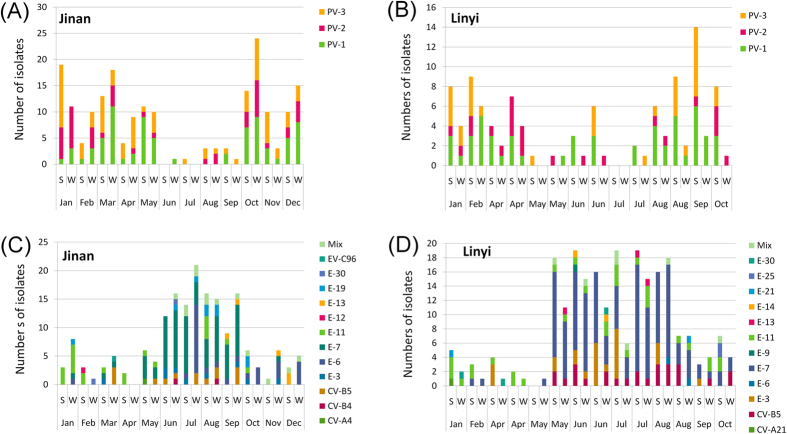
Monthly distribution of PVs (**A,B**) and NPEVs (**C,D**) in different samples collected in Jinan (**A,C**) and Linyi (**B,D**) in 2013. “S” and “W” in the X-axis are abbreviations for “Solids” and “Water”, respectively.

Typical summer-fall peak of detection of NPEV was observed in the monthly distribution of isolation in the cities of Jinan and Linyi (Fig. 2C,D), whereas the monthly distribution of PVs was different in that PV detection reached peak in spring and winter seasons (Fig. 2A,B).

### Different Adsorption to the Solids for E-3 and E-6

According to the one-year experience of surveillance, E-3 and E-6 has different preference to the solids and supernatant as described above. So, a test on their adsorption to the solids was performed to further ascertain whether this difference came from random error or did exist. After adding the viruses to sewage and stirring, both the viral titer left in sewage supernatant and the amount of virus adsorbed to the solids were examined (Table 2). Solids had stronger adsorption to E-3 proto and local strains, weaker to E-6 local strain, and moderate to PV-1, further demonstrating the difference in adsorption to the solids for different serotypes.

### Test of Adsorption to the Solids at Different pHs

The following tests in this study were performed in order to investigate the adsorption of enterovirus to the solids in different conditions. Sabin PV-1 was added to the inactivated sewage specimens with different pHs to a final titer of 362 50% tissue culture infective doses (TCID_50_)/100 μl. After thorough stirring of the sewage, the total titer and the titers of the supernatant and solids at different pHs were examined (Table 3). When pH = 1, 3, 11, or 13, the PV-1 titers were almost zero. When pH ranged from 5 to 9, acid medium facilitated the adsorption of virions to the solids, and in alkaline medium, virions preferred to exist in the supernatant. Hence, a slightly alkaline medium was suitable for eluting virions from the solids.

### Test of Adsorption to the Solids at Different Temperatures

PV-1 Sabin strain was seeded into inactivated sewage specimens to a final titer of 362 TCID_50_/100 μl. After stirring at 4 °C, 20 °C, and 37 °C respectively, the total titer and the titers of the supernatant and solids at different temperatures were examined (Table 4). Virions preferred to be adsorbed to the solids at 4 °C than those at 20 °C or 37 °C.

### Test of Adsorption to the Solids at Different Viral Titers

PV-1 was added to four sewage specimens with a final total titer ranging from 54 to 5792 TCID_50_/100 μl, of which 55.4% to 66.3% were adsorbed to the solids in the sewage (Table 5). A relative stable adsorption was observed at these four different titers. As generally viral titer in the sewage is low, and hardly exceeds the upper limit of 5792 TCID_50_/100 μl of this assay, it is reasonable to conclude that the proportion of virions adsorbed to the solids is similar for a single sewage sample.

### Test of Adsorption to the Solids at Different Solids Concentrations

According to the results of 25 sewage specimens collected in Shandong Province in 2013, the wet weight per sewage volume ranged from 0.45 to 1.86 g/l. So, in this adsorption and recovery test, the solids concentrations of 8 different inactivated sewage specimens were adjusted from 0.45 to 5.40 g/l, which covered the common situation in sewage surveillance.

**Figure 3:**
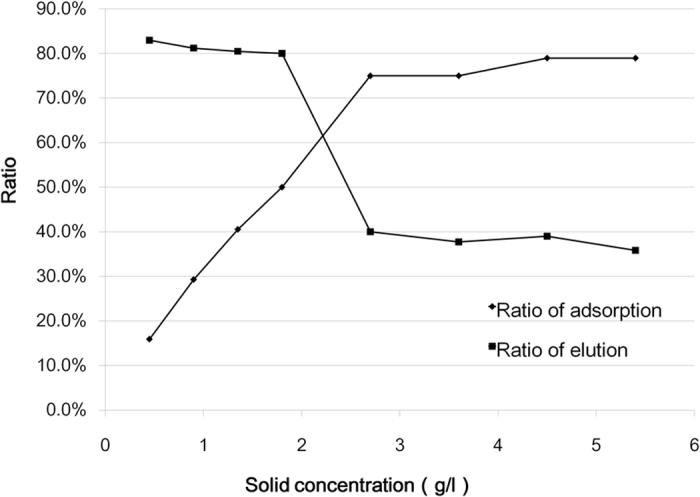
Influence of solids concentration on the PV-1 adsorption to the solids and elution. With the increase of the solids in the sewage, more PV-1 virions can be adsorbed on the solids. An abrupt decrease on elution ratio is presented when solids concentration ranges from 1.8 to 2.7 g/l.

PV-1 Sabin stain preparations were added to 8 sewage specimens with different solids concentration. The final total titer is 362 TCID_50_/100 μl. After stirring and titer, it was shown that with the increase of the solids in the sewage, more virions were adsorbed on the solids and less was left in the supernatant (Table 6, Fig. 3). When the solids concentration was more than 2.7 g/l, over 75% of seeded viruses were adsorbed on the solids. Within the range of actual solids concentration (0.45 to 1.80 g/l) in the sewage specimens in 2013, 15.90% to 49.99% of total viruses were adsorbed on the solids, and by using the elution method including a 3% beef extract solution at pH 9 and sonication, about 80.02% to 83.00% of the poliovirus virions adsorbed to the sewage solids can be recovered (Table 6).

## Discussion

Environmental surveillance has been used successfully in monitoring the circulation of PVs and NPEVs and it is of crucial importance in the final stage of the GPEI. In China, it is currently conducted in 9 provinces as a useful supplementary method for poliovirus surveillance, and all these experiences focus on the liquid fraction of the sewage^6^,^7^,^10^,^11^. So, it is necessary to ascertain the extent to which the enteroviruses were adsorbed to the solids in the sewage under different conditions.

This study provides comparative data from a one-year study in which enteroviruses in the solids and water of the sewage were investigated in parallel. Generally, the number of isolates from the solids was a little higher than that from water (Table 1). It should be noted that the method used for sewage water concentration is so called membrane adsorption/elution method which has been demonstrated of high efficiency^7^. So, the surveillance data suggest that it is valuable to analyze the solids in the sewage. Furthermore, adsorption bias was observed and confirmed for two serotypes of E-3 and E-6. Due to limited duration of this study, we cannot obtain enough data on the potential bias for other serotypes. Continued surveillance and comparison in the future might yield more interesting results.

According to the surveillance results in 2012, PV detection peaked in winter and spring, while in NPEV active seasons of summer and early autumn, PV detection decreased dramatically^7^. This distinction on seasonal distribution still appeared in this study in the two cities (Fig. 2). In our opinion, so called “musical chairs” might explain the occurrence of this phenomenon, at least partially. Human gut will not be occupied by NPEVs when they get quiet in winter and spring seasons, whereas routine OPV inoculation for children still maintains. Consequently, winter and spring turn out to be a good period for PV replication and transmission.

E-7 and CV-B5 turned out to be the most frequently isolated serotypes in 2013. Taking previous data into consideration, the annual most common serotypes varied in each year^7^. This may resulted from the epidemic pattern of temporal circulation for different serotypes^12^. Phylogenetic analysis revealed that these environmental sequences had high genetic diversity with foreign strains. CV-B5 associated meningitis outbreaks have occurred in Shandong Province in 2005 and 2009, respectively^9^. A close relationship was found between environmental CV-B5 and those meningitis strains, suggesting the possibility of occurrence of CV-B5 associated clinical cases in 2013. These results highlight the importance of environmental surveillance especially in regions with limited case-based surveillance.

In this study, the adsorption ratio under four most common conditions was investigated. First, similar to previous study^8^, adsorption to the solids was best achieved at low pH levels, and elution was practiced at pH 9.0. Higher pHs over 9.0 may inactivate the virus and is not suitable for elution. Second, temperature also influences the viral adsorption to the solids. Cold temperature (4 °C) facilitated the adsorption of virions to the solids. Third, as for the viral titer, our data suggested that alongside with the higher amount of virus in the sewage, the more virions were adsorbed to the solids, whereas the proportion of adsorption maintained stable for the same sewage specimen. Last, it is a very practical finding that the solids concentration in the sewage affected the adsorption dramatically. So, if high solids concentration is observed during the process of concentration, it is greatly suggested to analyze the virus adsorbed on the solids, otherwise the surveillance outcome simply focused on the supernatant is quite insufficient.

Many methods have been suggested for eluting enteroviruses from the solids or sludge including acid precipitation, organic flocculation, polyethylene glycol precipitation, etc^13^,^14^. These methods have some disadvantages that may detract from their usefulness and impair virus recovery effectiveness^13^. In this study, the elution method included a 3% beef extract solution at pH 9.0 and sonication, slightly modified from a previously described method which had demonstrated to yield higher viral titers than others^14^,^15^. In a preliminary test on the sonication time, enterovirus titer in elution solution maintained almost unchanged if undergoing sonication within 3 minutes, while decreased if sonication over 3 minutes (data not shown). So, the duration for sonication was set as 3 minutes. The high elution rate of more than 80% for common sewage samples demonstrated the high efficiency of this method.

The data from adsorption and elution test and surveillance experience in this study are based on the experiment using local sewage samples. As the sewage constitution is complicated and may vary in different regions, it is necessary to get samples from other areas to validate these results. Nevertheless, it is reasonable to conclude the conditions investigated in this study may affect the EV adsorption to the solids in the same way. Also, commercially produced beef extracts are a variable commodity. Its natural pH is variable and should be adjusted to 9.0 if used for elution. Otherwise virus inactivation or less eluting efficiency will be resulted at higher or lower pHs, respectively.

In conclusion, the EV adsorption to the solids under different conditions was investigated, and one-year experience of surveillance was described. The results of this study suggest that the outcome from the supernatant and solids should be incorporated in enterovirus environmental surveillance to better understand the local circulation of different serotypes.

## Methods

### Sewage Sampling and Processing in Environmental Surveillance

Jinan is the capital city, and Linyi is the largest city in Shandong Province. Two waste water treatment plants from the cities of Jinan and Linyi separately were selected as the sampling sites. Each plant covers about half of the metropolitan area of each city. Sewage samples were collected every month from January to December in 2013. More than 1 l of sewage was collected by grab sampling method and transported to the laboratory on ice. One liter of the sewage was centrifuged at 3000 × *g* for 30 min at 4 °C. Then the supernatant and the sediments were processed in parallel.

The supernatant was concentrated by using the membrane adsorption/elution method as described previously^16^,^17^. Briefly, MgCl_2_ was added to a final concentration of 0.05 M, and the pH was adjusted to 3.5. Then the solution was filtered through a 0.45 μm mixed cellulose ester membrane filter (ADVANTEC, Japan). Absorbed viruses on the filter were then eluted with 10 ml 3% beef extract solution followed by ultrasonication for 3 min. After centrifugation at 3000 × *g* for 30 min, the supernatant was filtered through a 0.22 μm filter and was ready for cell inoculation.

The method from eluting enteroviruses from the solids is slightly modified as described previously^14^,^15^: Briefly, sewage was centrifuged at 3000 × *g* for 30 min at 4 °C. The sediments were re-suspended in 10 ml of the 3% beef extract solution (pH = 9.0), followed by ultrasonication (50 W, 20 kHz) for 3 min on ice. After centrifugation at 12000 × *g* for 5 min at 4 °C, the supernatant was filtered through a 0.22 μm filter and its pH was adjusted to 7 by 0.5 M hydrochloric acid.

### Virus Isolation

L20B, RD and HEp-2 cell lines were used for enterovirus isolation as suggested by WHO^18^,^19^. Cells were seeded in each tube with an amount of 1 × 10^5^. For each cell line, each sewage sample and each origin (solids or supernatant), 18 parallel cell vials with standard monolayer cell culture were inoculated with 200 μl of concentrated solution for each vial.

### VP1 Amplification and Sequencing

VP1 sequencing was performed on all the EV isolates. Total RNA was extracted from 140 μl of the viral isolates using QIAamp viral RNA mini kit (Qiagen, USA).One step reverse transcription-PCR (RT-PCR) was performed using Access RT-PCR System (Promega, USA). Primer pair UG1/UC11^20^ was used to amplify the entire VP1 coding region of PV isolates. Primer pair 187/011^21^ that corresponds to the 3′ end of VP1 and 5′ end of the 2A was used for amplification of an about 750-nucleotide sequence of EV-B strains. Primer pairs 486/488^22^ and 040/011^21^ were used for amplification of entire VP1 sequence of EV-A strains. Primer pairs UF1/UR1 and 040/DR1 were used for the amplification of entire VP1 sequence of EV-C strains^21^,^23^. Blank control, negative control and positive control were included in the RT-PCR reaction. PCR positive products were purified and sequenced bi-directionally with the BigDye Terminator v3.0 Cycle Sequencing kit (Applied Biosystems), and sequences were analyzed by ABI 3130 genetic analyzer (Applied Biosystems).

### Sequence Analysis and Statistical Analysis

Molecular typing based on VP1 sequences was performed by using online Enterovirus Genotyping Tool version 0.1^24^. Nucleotide sequence alignments were carried out by BioEdit 7.0.5.3 software^25^. Phylogenetic trees were constructed by Mega 5.0^26^ using neighbor-joining method after estimation of genetic distance using the Kimura two-parameter method^27^. In order to reduce computational load, the 100% identical sequences were manually removed. Wilcoxon signed ranks test was performed via SPSS 17.0 software to investigate the possible difference on numbers of isolates between the two origins.

### Sewage used for the adsorption tests

Sewage specimens used for the adsorption and recovery tests was collected from the inlet collector canal of the sewage treatment plant in Jinan. After microorganisms in the sewage were inactivated at 56 °C for 30 min by water bath, the sewage was cooled to room temperature and was ready to be used in the following adsorption tests.

### Test of Adsorption to the Solids for E-3 and E-6

Inactivated sewage specimen was divided into 4 aliquots of 500 ml each, and the wet weight of the solids per sewage volume was 0.9 g/l. Preparations of E-3 prototype stain Morrisey, E-3 local strain JN12010 (Wang *et al*.^7^), E-6 local strain JN111219-1 (Tao *et al*.^6^), and PV-1 Sabin strain were added into separated sewage samples with a theoretical final titer around 256 TCID_50_/100 μl. Also, a parallel of sewage supernatant was added with different viruses as control. After mixing for 1 h on a magnetic stirrer at room temperature, the sewage aliquots were centrifuged at 3000 × *g* for 30 min at 4 °C. The titers of the supernatants and the controls were examined. Briefly, the serial four-fold dilution was prepared with MEM, and 100 μl of each was transferred to the RD cell monolayer in a microplate. The titer (TCID_50_/100 μl) was determined by observing cytopathic effect (CPE) microscopically after 5 days. The microtiter assays were performed triplicate.

### Test of Adsorption to the Solids at Different pHs

Inactivated sewage specimen was divided into 7 aliquots of 500 ml each, whose pHs were adjusted to 1, 3, 5, 7, 9, 11, and 13, respectively by 0.5 M hydrochloric acid or sodium hydroxide solution. The wet weight of the solids per sewage volume was 0.9 g/l. PV-1 Sabin strain was seeded into these sewage aliquots to a theoretical final titer of 362 TCID_50_/100 μl. The following stirring, centrifugation, and titer in triplicate were performed as described above.

Also, the pHs of 7 inactivated sewage supernatants were adjusted to 1, 3, 5, 7, 9, 11, and 13, and the virus was added to them in parallel. After stirring, their titers were examined and served as the total viral titer added in each sewage aliquot.

### Test of Adsorption to the Solids at Different Temperatures

Inactivated sewage specimen was divided into 3 aliquots of 500 ml each. PV-1 Sabin strain was seeded into these sewage specimens to a final titer of 362 TCID_50_/100 μl. After mixing for 1 h on a magnetic stirrer at 4 °C, 20 °C, and 37 °C respectively, the sewage aliquots were centrifuged at 12000 × *g* for 1 min at respective temperatures. Then the titers of the supernatants and the controls were examined. The microtiter assays were performed triplicate.

### Test of Adsorption to the Solids at Different Viral Titers

The inactivated sewage specimen was divided into 4 aliquots of 500 ml each. The wet weight of the solids per sewage volume was 0.9 g/l. PV-1 Sabin strain was seeded into the sewage aliquots to a theoretical final titer from 50 to 5000 TCID_50_/100 μl. The following stirring, centrifugation, titer in triplicate, and sewage supernatant control were performed as described above.

### Test of Adsorption to the Solids at Different Solids Concentrations

The inactivated sewage specimen was settled at 4 °C for one day, and then the sediments were centrifuged at 3000 × *g* for 30 min at 4 °C. The solids were weighed and re-suspended in different volumes of sewage supernatant to prepare 8 sewage specimens with different solids concentrations, ranging from 0.45 to 5.40 g/l. This range covers the common situation in sewage surveillance. PV-1 Sabin strain was seeded into these sewage specimens to a final titer of 362 TCID_50_/100 μl. The following stirring, centrifugation, titer, and sewage supernatant control were performed as described above.

### Nucleotide Sequence Accession Numbers

The VP1 nucleotide sequences of environmental isolates described in this study were deposited in the GenBank database under the accession numbers KU049787 to KU050050.

## Additional Information

**How to cite this article**: Tao, Z. *et al*. One-year Survey of human enteroviruses from sewage and the factors affecting virus adsorption to the suspended solids. *Sci. Rep.* **6**, 31474; doi: 10.1038/srep31474 (2016).

## Figures and Tables

**Table 1:** Number of isolates of different serotypes from environmental surveillance in 2013 and the comparison between the solids and supernatant origin.

Serotype	No. of isolates from all sewage samples			Comparison of no. of isolates from the solids and supernatant of 26 sewage samples				
	City		Sum	Sewage component		Sum	Wilcoxon Signed Ranks Test	
	Jinan	Linyi		Solids	Supernatant		Z	*P*
PV-1	90	55	145	72	58	130	1.502	0.133
PV-2	53	23	76	29	40	69	1.216	0.224
PV-3	82	31	113	69	36	105	1.882	0.06
CV-A4	1	0	1	1	0	1		
E-3	9	26	35	30	5	35	2.751	0.006*
E-6	36	4	40	4	27	31	2.384	0.017*
E-7	96	163	259	107	94	201	0.803	0.422
E-9	0	1	1	1	0	1		
E-11	26	33	59	32	19	51	1.388	0.165
E-12	2	0	2	1	0	1		
E-13	5	3	8	4	4	8		
E-14	0	2	2	1	1	2		
E-19	10	0	10	4	5	9		
E-20	0	2	2	0	0	0		
E-21	0	2	2	1	1	2		
E-25	0	3	3	2	0	2		
E-30	2	3	5	0	5	5		
CV-B3	1	0	1	0	0	0		
CV-B4	3	0	3	0	2	2		
CV-B5	22	55	77	16	24	40	1.153	0.249
CV-A21	1	1	2	1	0	1		
EV-C96	1	0	1	0	1	1		
Mix^a^	15	11	26	11	9	20		
Total EV	455	418	873	386	331	717		

^*^Indicates a *P* value less than 0.05.

^a^“Mix” indicates NPEV isolates that could not be serotyped by RIVM antibody pools, and VP1 sequencing revealed mixed peaks.

**Table 2:** Test on adsorption of PV-1, E-3 and E-6 to the solids.

Serotype/Strain	Titer (TCID_50_/100 μl)			Ratio of solids adsorption
	Total	Supernatant	Solids	
PV-1/Sabin	304	215	89	29.3%
E-3/Morrisey	256	76	180	70.3%
E-3/ JN12010	256	54	202	78.9%
E-6/ JN111219-1	256	239	17	6.6%

**Table 3:** Adsorption of PV-1 to the solids at different pHs.

pHs	Titer (TCID_50_/100 μl)			Adsorption ratio of the solids
	Total	Supernatant	Solids	
1	0	0	0	/*
3	0	0	0	/*
5	362	0	362	100%
7	362	128	234	64.6%
9	362	256	106	29.3%
11	1.19	0	1.19	/*
13	0	0	0	/*

^*^Virions were hardly detected probably due to inactivation at these pHs.

**Table 4:** Adsorption of PV-1 to the solids at different temperatures.

Temperatures	Titer (TCID_50_/100 μl)			Adsorption ratio of the solids
	Total	Supernatant	Solids	
4 °C	362	92	270	74.6%
20 °C	362	220	142	39.2%
37 °C	362	208	154	42.5%

**Table 5:** Adsorption of PV-1 to the solids at different viral titers.

Expt no.	Titer (TCID_50_/100 μl)			Adsorption ratio of the solids
	Total	Supernatant	Solids	
1	5792	1952	3840	66.3%
2	1152	514	638	55.4%
3	234	104	132	56.4%
4	54	20	34	63.0%

**Table 6:** Adsorption and elution of PV-1 on the solids at different solids concentration.

Sewage no.	Solids concentration (g/l)	Titer (TCID_50_/100 μl)			Ratio (%)	
		Supernatant	Adsorption to the solids	Elution from the solids	Adsorption to the solids	Elution from the solids
1	0.45	304.4	57.6	47.8	15.90	83.00
2	0.90	256.0	106.0	86.1	29.28	81.23
3	1.35	215.3	146.7	118.2	40.53	80.50
4	1.80	181.0	181.0	144.8	49.99	80.02
5	2.70	90.5	271.5	108.6	75.00	40.00
6	3.60	90.5	271.5	102.4	75.00	37.72
7	4.50	76.1	285.9	111.5	78.98	39.00
8	5.40	76.1	285.9	102.4	78.98	35.82
